# Application of contrast-enhanced ultrasound to improve the management of microwave thyroid nodule ablation

**DOI:** 10.1177/1742271X261417150

**Published:** 2026-02-15

**Authors:** Gibran Timothy Yusuf, Husam Wassati, Sudeendra Doddi, Gabriele Galata, Paul S Sidhu

**Affiliations:** 1 King’s College Hospital, London, UK; 2 Princess Royal University Hospital, London, UK

**Keywords:** CEUS, thyroid, ablation, ultrasound

## Abstract

Thermal ablation has gathered population for treatment of benign thyroid nodules in recent years as an alternative to surgery. This utilises techniques such as radiofrequency, microwave and laser ablation. Contrast-enhanced ultrasound has also gained widespread use over the last few decades and offers a number of advantages in intervention. This article will describe the utility of contrast-enhanced ultrasound in microwave thyroid ablation from a pre-procedure, intraprocedural and post-procedural perspective. The application of contrast-enhanced ultrasound allows for a more comprehensive management strategy for thyroid nodule ablation.

## Introduction

### Incidence and risk stratification

Thyroid nodules are particularly common and have been increasingly recognised in the population with a reported presence in up to 60% of the adult population.^1^ Risk stratification tools are used to determine benign from malignant features on ultrasound. Traditionally both malignant and symptomatic benign nodules were treated by surgical excision. There has been no significant increase in mortality associated with thyroid cancer for a number of years, and it is widely recognised that the majority of thyroid nodules are benign.^2^ It is also considered that most thyroid malignancy is indolent and could undergo conservative management.^3^ Ultrasound is the mainstay for thyroid nodule evaluation given the excellent spatial resolution, in the United Kingdom this is by means of the BTA (British Thyroid Association)^4^ guidelines with a number of further similar systems existing globally such as TIRADS from the American College of Radiologists.^5^ Key features are used to determine the risk of thyroid malignancy from nodule characteristics and therefore the need for cytological assessment influencing further management. Despite the benign nature of many thyroid nodules, they may still require treatment. Benign nodules exhibit a wide range of symptoms from localised mass effect on the trachea or oesophagus, but frequently the patient concern is the cosmetic aspect, potentially psychological symptoms and a residual scar following surgery is an issue for many patients. Furthermore, symptoms may be difficult to recognise given they may be positional, mild severity or patients can develop adaptations to their life in order to cope.

### Management strategies

Surgery is considered the mainstay of treatment for malignancy, and for symptomatic benign disease in the form of total or hemithyroidectomy, which carries a significant risk, hospital stay and scarring over the neck.^6^ While surgery has been the procedure of choice for the management of thyroid cancer, recent reports demonstrate the utility of ablation therapies in the treatment of small papillary thyroid cancers.^7^ The management of benign thyroid nodules remains predominantly surgical, but with the known patient morbidity^8^ is often not the preferred option for the patient. The high numbers of patients with symptomatic benign thyroid nodules, who are either considered unsuitable for traditional surgical removal or do not wish to undergo it means that ablative therapy may be a preferable option.

### Overview of microwave thyroid ablation

Ablation of thyroid nodules has developed as a technique using ethanol or thermal energy.^2,9,10^ Ethanol ablation is considered the most effective percutaneous treatment for cystic nodules but is less suitable for solid lesions due to the unpredictable diffusion.^9^ Thermal ablation is a more recent technique and exists in several forms, originating from radiofrequency ablation (RFA), to now include microwave and laser ablation as well as early work with cryoablation.^10^

Microwave ablation (MWA) uses electromagnetic energy resulting in the generation of heat to destroy tissue and is used extensively elsewhere within the body, including the liver, lung, kidney and soft tissue where it is predominantly considered a curative cancer treatment. This is in distinction to the benign utility in the thyroid,^2,11^ although a growing body of work is developing for small volume malignant disease.^7,10,12–14^ Several techniques have been proposed for microwave ablation, including a traditional moving shot technique where the antenna is slowly withdrawn along multiple lines of the nodule, and the multiple overlapping technique creating several distinct zones of ablation which overlap to encompass the entire nodule. More recently proposed, using low power uncooled microwave technology, is the fluid motion technique which involves continuous movement resulting in both thermal and mechanical disruption of the nodule.^15^ A recent study compared MWA and RFA with non-inferiority and MWA achieving 80% volume reduction ratio (VRR) at two years.^16^ The multiple overlapping ablation technique achieves similar outcomes at two years while the fluid motion technique demonstrated 81% VRR at six months.^15,17^ It has been appreciated that there are independent risk factors for efficacy of microwave ablation, including maximum nodule diameter, preoperative volume, internal architecture and the appearance immediately post ablation^18^

### Contrast-enhanced ultrasound in thyroid nodules

Contrast-enhanced ultrasound (CEUS) is a well-recognised technique using microbubbles to delineate macro and microvascular structures as a true blood pool agent. The microbubbles consist of an inert gas (sulphur hexaflouride) encased in a phospholipid shell administered intravenously with transpulmonary stability. This combines the excellent spatial resolution of ultrasound with dynamic vascular assessment not otherwise possible in real time. The contrast agent is very safe and is not nephrotoxic or hepatotoxic negating the needs for prior blood tests.^19–22^ In addition, there are benefits conventionally associated with ultrasound such as the absence of ionising radiation, portability to perform by the bedside as well as the real time nature allowing accurate guidance of interventional procedures. CEUS has a myriad of uses, most commonly recognised for the characterisation of focal liver lesions but also includes renal, testicular, vascular and interventional uses. Guidelines have been released for its use from the European Federation Society of Ultrasound Medicine and Biology detailing both the hepatic and non-hepatic uses.^19,20^

The role of vascularity assessment in thyroid nodules has been debated and the use of Doppler has been equivocal with inclusion in the BTA (British Thyroid Association guidelines U classification) but absent in ACR (American College of Radiology,–TIRADS).^4,5^ It is felt there is wide overlap with benign and malignant disease in Doppler evaluation, and by virtue of this the role of CEUS is debated and the most recent EFSUMB (European Federation of Ultrasound in Medicine and Biology) guidelines suggests a role in research only.^19^ However there have been a number of themes which have emerged with dynamic vascular assessment. A recent study evaluated the addition of CEUS to TIRADS and demonstrated the reduction in the number of unwarranted FNA as well as the highest biopsy yield of malignancy when compared to a number of scoring systems including TIRADS.^23^ Hypoenhancement and heterogeneous enhancement are associated with malignancy while a residual hyperenhancing rim of a nodule has been shown to 100% specific for benignity.^23,24^

This review aims to delineate the role of CEUS to aid thyroid ablation, including prior to the procedure, intraprocedural and post procedure. No ethical approval was required for this review article.

### Pre-procedure

**Figure 1.**
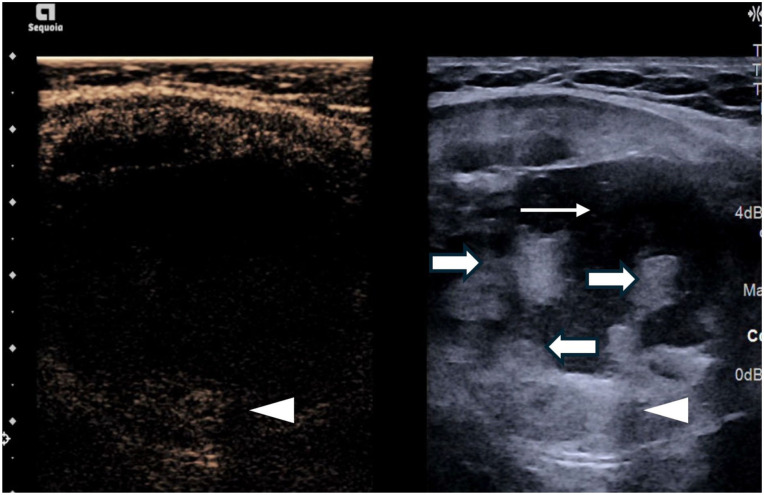
Simultaneous greyscale and contrast-enhanced ultrasound image showing non-enhancement of the majority of solid components (thick arrow) indicating retracted clots with a small enhancing peripheral solid component (arrowhead) and central fluid portion (thin arrow).

Preprocedural assessment can be performed diagnostically as outlined above but also has a role in the immediate assessment before ablation. CEUS also allows identification of truly vascularised areas of the nodule which Doppler may not appreciate. CEUS has been shown to be useful in order to better define solid tissue and low level internal vessels.^25^ Conversely CEUS can help define haemorrhagic cysts which may mimic solid lesions (Figure 1). Furthermore aspiration of cystic areas by CEUS targeting, prior to ablation of solid components allowing for improved access and avoidance of fluid leakage. By comparison if a nodule is purely cystic, ablation with ethanol has been proven to be a therapeutically and financially effective technique compared to thermal ablation.^2,9,26^

Avoidance of major vasculature is of particular importance in thyroid ablation. The macrovasculature in the perithyroidal region (particularly arterial supply) can be better visualised on CEUS and may influence approach taken to avoid inadvertent vascular injury. It has been shown elsewhere in the body that a significant number of procedures change trajectory based on procedural CEUS in 48% of cases.^27^

**Figure 2.**
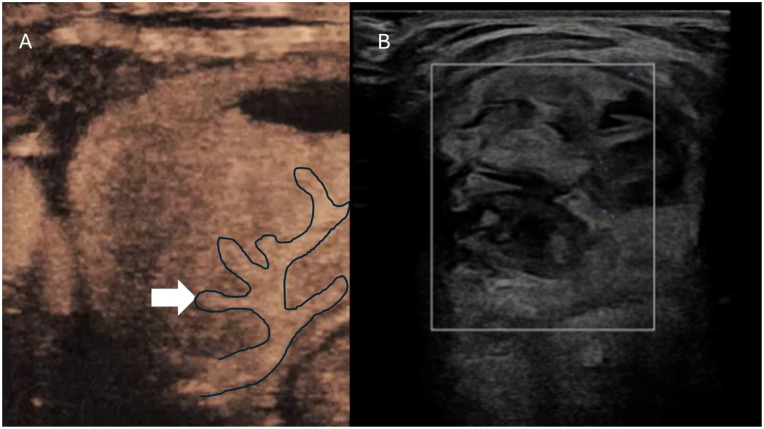
(a) Contrast-enhanced ultrasound image demonstrating a feeding artery centrally within a thyroid nodule pre ablation. (b) Post ablation microvascular Doppler showing no signal within the nodule and the feeding artery.

While avoidance of major extrathyroidal vasculature is critical, particular techniques have evolved to target a nodules major arterial and venous supply. The artery first technique involves placement of the ablation probe into the centre of a feeding artery in order devascularise the nodule, encouraging better treatment of the periphery which may be difficult otherwise to access (Figure 2). This is considered particularly useful in larger nodules where up to 50% demonstrate a central vascular pattern compared to 15% in smaller nodules.^28^ The artery first technique with RFA showed significant improvement in VRR at three months.^29^ MWA showed significant reduction in the VRR within the first three months as well as at six months.^29^ The use of CEUS allows for clear depiction of feeding artery borders as well as the absent flow when treated, which may be difficult to evaluate in the presence of artefact generated from ablation or the low flow state, on Doppler imaging alone. The degree of nodular regrowth has shown to be less when CEUS is combined with artery first ablation.^30^ Marginal vein ablation is considered also an effective method to interrupt the draining veins and thought to improve infarction and necrosis of peripheral areas and decreased regrowth rates.^31^ When described with RFA, there is visualisation of gas bubbles filling the vein which can potentially impair assessment of the remaining nodule perfusion indicating the further role for CEUS to highlight residual areas of viable tissue.

### Intra-procedure

**Figure 3.**
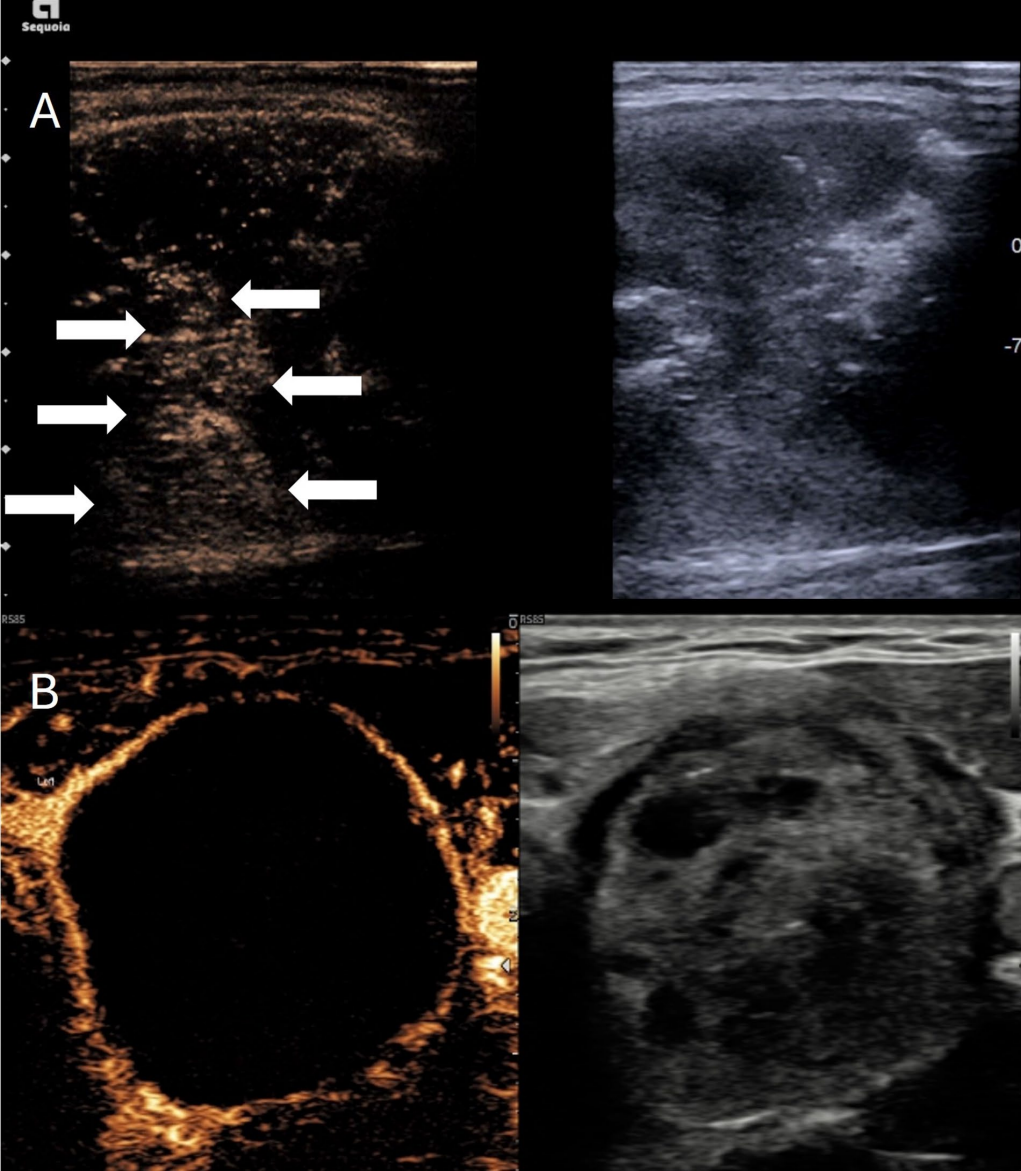
(a) Simultaneous greyscale and contrast-enhanced ultrasound image after microwave thyroid ablation. The greyscale image shows a majority hypoechoic nodule with some echogenic regions immediate post ablation. Contrast-enhanced ultrasound shows residual enhancing tissue centrally (arrows) despite the hypoechoic appearance indicating viable tissue needing retreatment. (b) A second patient with simultaneous greyscale and contrast-enhanced ultrasound showing complete ablation of the nodule without any internal enhancement.

CEUS is often performed immediately prior to the procedure and can aid in depiction of degree of enhancing tissue as described, but also allows visualisation of the macrovasculature. However, the main application during the ablation procedure is to identify and quantify the volume of ablated tissue.^25^ Ablation of benign disease differs from that of malignant disease and the ablation zone typically remains within the margins of the nodule as opposed to beyond it. As a result regrowth of nodules is possible with 1.35% estimated in long terms studies,^32,33^ and it is of importance to ensure sufficient ablation has occurred. Thermal ablation generates gas obscuring marginal visualisation; this occurs less and clears quickly with low energy microwave ablation. The rapid clearance of echogenic artefact in low energy microwave ablation may mean the extent of effective ablation can be difficult to judge, particularly in large nodules. The ability to image the microvasculature to a capillary level ensures the amount of residual tissue can be assessed and repeat ablation performed if needed (Figure 3). This principle was shown in a study comparing ablation with and without CEUS and compared to MRI quantification. The CEUS group was shown to have a significantly higher necrosis rate (97 vs 86%) compared to conventional ultrasound guidance which resulted in an improved reduction in volume.^33^ A study by Zhang et al. demonstrated that contrast-enhanced ultrasound led to a more complete degree of ablation compared to conventional guidance. Statistical significance was also obtained for volume reduction at 1, 3, 6 and 12 months with statistically decreased recurrence rate and complications.^34^

**Figure 4.**
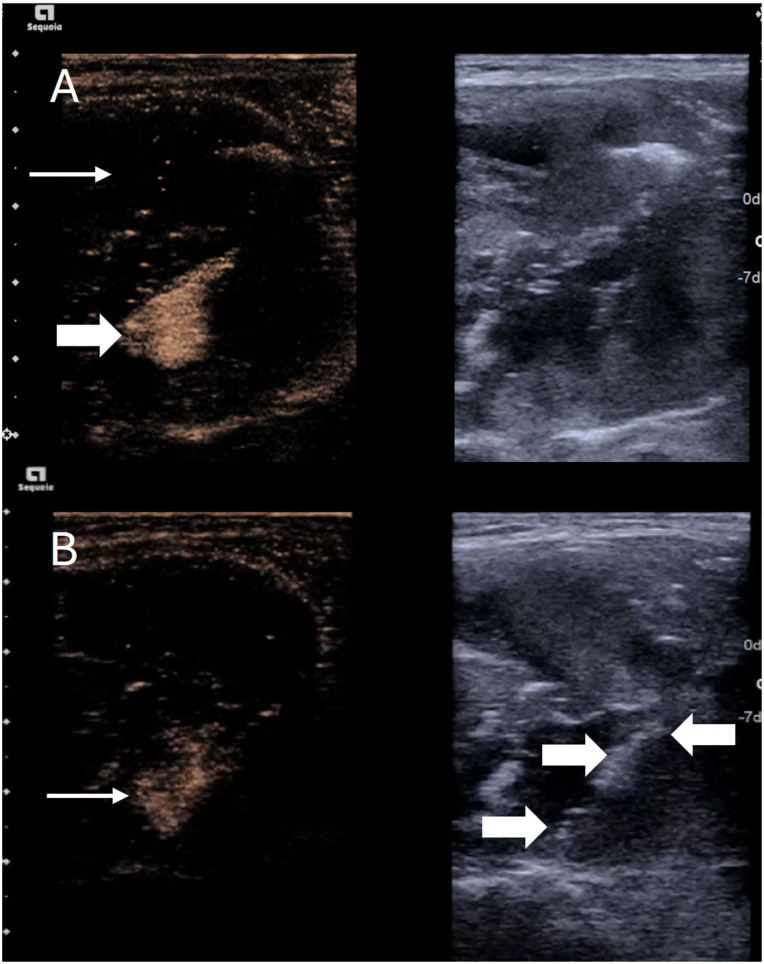
Simultaneous greyscale and contrast-enhanced ultrasound images of a thyroid nodule post microwave ablation. (a) Immediate post ablation an area of active contrast extravasation is seen in keeping with intranodular arterial haemorrhage (thick arrow) within an otherwise fully ablated nodule. (b) The microwave probe was placed at the point of haemorrhage (thick arrows) and there was slowing of the extravasation and eventual cessation.

**Figure 5.**
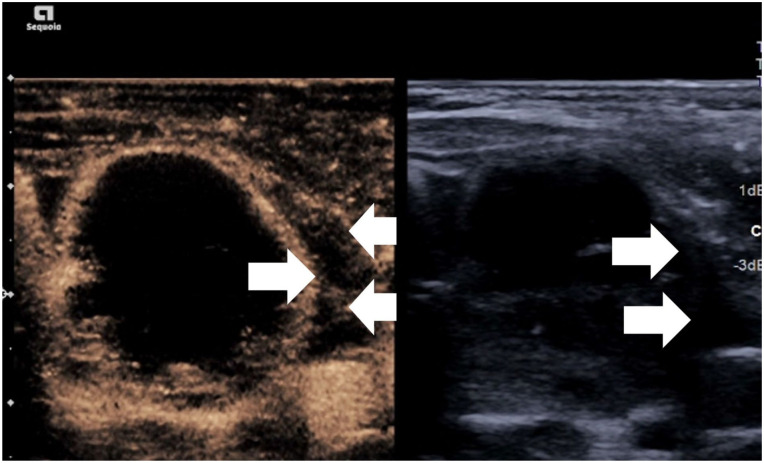
Simultaneous greyscale and contrast-enhanced ultrasound image showing satisfactory ablation of the thyroid nodule but a small avascular echogenic haematoma in the lateral perithyroidal region (arrows).

Within the procedure it is also possible to assess for complications. Haemorrhage can occur within a nodule from transection of friable vessels or incomplete coagulation. A consequence of this is increased intranodular volume and potentially nodule rupture. Clinical manifestation of nodule rupture can be dramatic with prominent neck swelling and localised bruising, but may present with airway compression or fistulation. While most nodule ruptures are able to be managed conservatively, some may require drainage or thyroidectomy.^35^ Post ablation CEUS can identify active haemorrhage or pseudoaneurysm formation which may result in delayed rupture. This is particularly apparent on a background of an ablated nodule where the contrast is seen to pool in a slow expansile, amorphous pattern. Due to the heat generation, the microwave antenna can be placed in contact with the point of bleeding and used to coagulate the offending vessel (Figure 4). Cystic or spongiform nodules may seep fluid from the access point of the nodule and when combined with blood there may be increased echogenicity making it difficult to assess a perithyroidal haematoma. The avascular nature of the haematoma is in contrast to the uptake of the surrounding tissues on CEUS (Figure 5) allowing clear visualisation. Conversely there can also be early identification of active extrathyroidal haemorrhage if contrast is seen within the collection. This technique has been used in other interventional procedures to identify echogenic collections to facilitate early drainage or cessation of haemorrhage.

### Post-procedure

**Figure 6.**
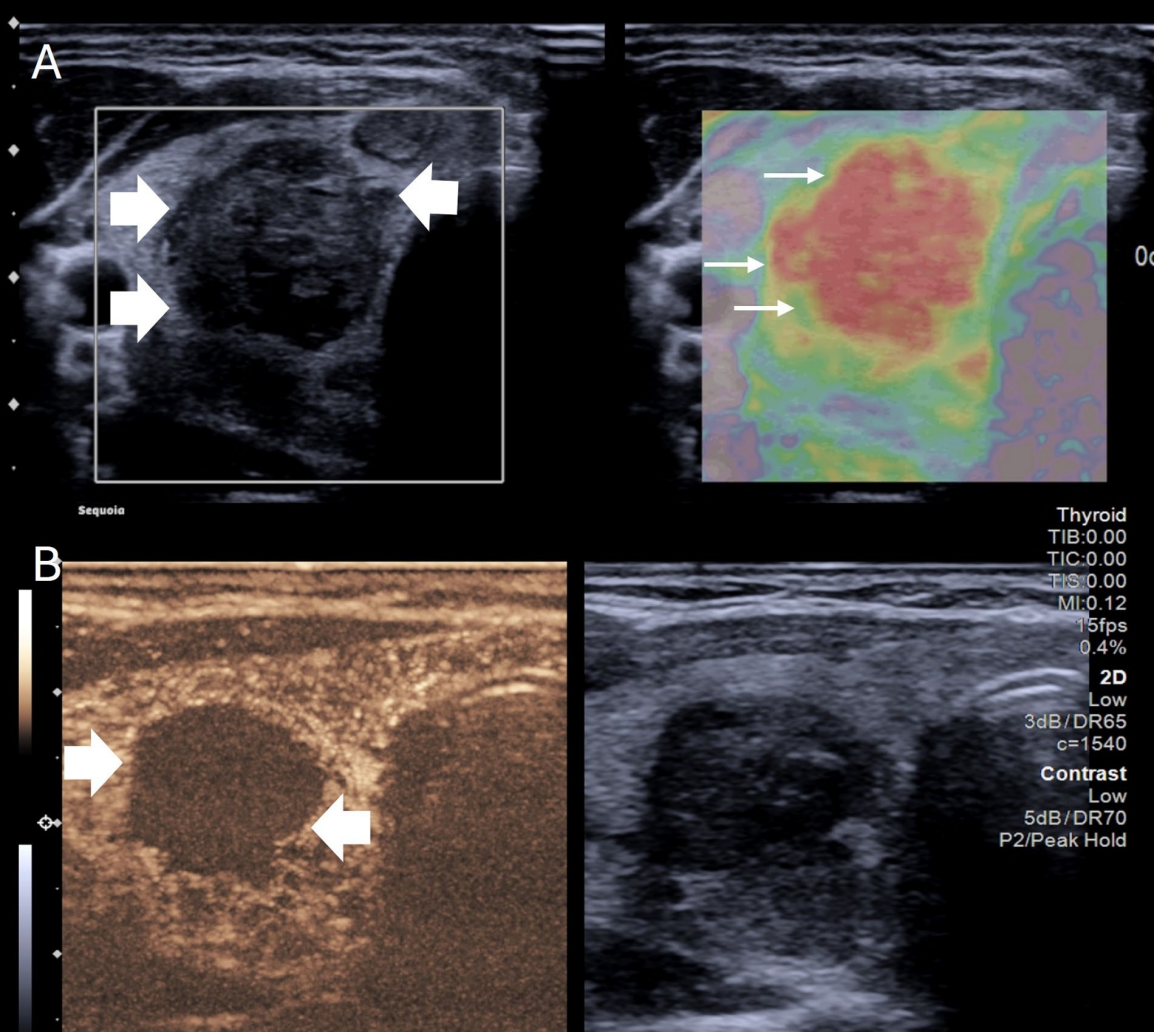
Three-month follow-up of a thyroid nodule post ablation. (a) simultaneous b mode and strain elastography image showing a hypoechoic, irregular (thick arrows), stiff nodule (thin arrows) which would be suspicious for malignancy in the absence of prior history of ablation. (b) Simultaneous B mode and contrast-enhanced ultrasound image of the same patient which shows no enhancement (thick arrows) within the nodule reassuring of effective ablation and a benign lesion.

As described in the immediate post ablation period, CEUS can assess for complications and adequacy of ablation but can also be used in later follow-up. On follow-up it has been noted that the ablated nodule can mimic malignancy on greyscale ultrasound. In particular ablated nodules can appear to have irregular borders, markedly hypoechoic nature and potentially have calcification.^36^ The wide overlap of Doppler signal in malignancy and benign nodules can make evaluation difficult particularly as these nodules also often appear hard on elastography, an ultrasound-based method for assessment of stiffness of a lesion. Similar appearances are also seen with cystic nodules which have undergone ethanol ablation, so-called mummified nodules. Critically these remain non-vascular due to the retracted devascularised nature, as a result contrast-enhanced ultrasound demonstrating avascularity is of critical reassurance (Figure 6). This may be especially important if the patient is not followed up at the institution at which the ablation was performed. The authors postulate that CEUS may be an important tool in the evaluation of suspected previously ablated nodules. Conversely if a nodule is incompletely ablated and the patient remains symptomatic, contrast-enhanced ultrasound can identify areas to address for retreatment if not readily identifiable on greyscale imaging. It is also notable that the margins of the nodule postablation are not always obvious. Yan et al. demonstrated that CEUS provided good evaluation between observers of the ablation zone. Significantly the volume of the ablated area was consistently larger when measured by greyscale ultrasound compared to CEUS.^37^ Findings are also shown by Schiaffino et al.^38^ where there was significantly higher inter and intraobserver variability as well as reproducibility in the follow-up period.

## Conclusion

Contrast-enhanced ultrasound provides an accurate and safe method of micro and macrovascular depiction within the thyroid gland. While there is utility in the preprocedural assessment for diagnostic purposes, the immediate preprocedural assessment with CEUS defines the best approach for access as well as adequacy of ablation and identifying complications. Post treatment CEUS also allows immediate and later evaluation of ablated tissue and prognostic value. Furthermore, post ablation nodules may appear as malignant on greyscale ultrasound and CEUS allows reassurance of a completely avascular lesion avoiding misinterpretation.
